# *C. elegans* ATAD-3 Is Essential for Mitochondrial Activity and Development

**DOI:** 10.1371/journal.pone.0007644

**Published:** 2009-10-30

**Authors:** Michael Hoffmann, Nadège Bellance, Rodrigue Rossignol, Werner J. H. Koopman, Peter H. G. M. Willems, Ertan Mayatepek, Olaf Bossinger, Felix Distelmaier

**Affiliations:** 1 Department of General Pediatrics, University Children's Hospital, Heinrich-Heine-University, Düsseldorf, Germany; 2 Institute for Genetics, Heinrich-Heine-University, Düsseldorf, Germany; 3 Institut National de la Santé et de la Recherche Médicale (INSERM), U688 Physiopathologie Mitochondriale, Universite Victor Segalen-Bordeaux 2, Bordeaux, France; 4 Department of Biochemistry, Nijmegen Center for Molecular Life Sciences, Radboud University Nijmegen Medical Centre, Nijmegen, The Netherlands; 5 Microscopical Imaging Center, Nijmegen Center for Molecular Life Sciences, Radboud University Nijmegen Medical Centre, Nijmegen, The Netherlands; 6 Institute of Molecular and Cellular Anatomy, RWTH Aachen University, Aachen, Germany; Baylor College of Medicine, United States of America

## Abstract

**Background:**

Mammalian ATAD3 is a mitochondrial protein, which is thought to play an important role in nucleoid organization. However, its exact function is still unresolved.

**Results:**

Here, we characterize the *Caenorhabditis elegans* (*C. elegans*) ATAD3 homologue (ATAD-3) and investigate its importance for mitochondrial function and development. We show that ATAD-3 is highly conserved among different species and RNA mediated interference against *atad-3* causes severe defects, characterized by early larval arrest, gonadal dysfunction and embryonic lethality. Investigation of mitochondrial physiology revealed a disturbance in organellar structure while biogenesis and function, as indicated by complex I and citrate synthase activities, appeared to be unaltered according to the developmental stage. Nevertheless, we observed very low complex I and citrate synthase activities in L1 larvae populations in comparison to higher larval and adult stages. Our findings indicate that *atad-3(RNAi)* animals arrest at developmental stages with low mitochondrial activity. In addition, a reduced intestinal fat storage and low lysosomal content after depletion of ATAD-3 suggests a central role of this protein for metabolic activity.

**Conclusions:**

In summary, our data clearly indicate that ATAD-3 is essential for *C. elegans* development *in vivo*. Moreover, our results suggest that the protein is important for the upregulation of mitochondrial activity during the transition to higher larval stages.

## Introduction

Mitochondria are semi-autonomous organelles in eukaryotic organisms, which have a profound role in cell viability through the control of energy production via oxidative phosphorylation (OXPHOS) [1]. It is generally believed that mitochondria originated in ancestral eukaryotic cells through endosymbiosis of aerobic bacteria [2]. While the control of most of the mitochondrial proteins is executed by the nuclear DNA, mitochondria still retain their own genome as well as facilities for multiplying themselves by fission and fusion events.

The mitochondrial DNA (mtDNA) typically forms a circular, double-stranded molecule that spans about 16,569 base pairs. It encodes 37 genes, all of which are essential for normal mitochondrial function (13 genes encode OXPHOS proteins, 22 encode transfer RNAs, and two encode subunits of ribosomal RNA) [3]. Most mammalian cells contain between 1000 and 10,000 copies of mtDNA. There are much higher copy numbers (∼175,000 copies) in mature oocytes [4], which may reflect that a sufficient mtDNA reservoir is required to ensure distribution of mitochondria to the cells of the early embryo. This also holds true for proliferating cells where the number of mitochondria and mtDNA molecules is approximately doubled before dividing, providing equal amounts to its daughter cells [5].

Obviously, gene expression and (timely) replication of mtDNA require a great number of regulatory factors, which are nuclear-encoded and translocate to the mitochondria to execute their tasks. In addition, specific proteins are involved in maintenance and structural organization of mtDNA. A novel candidate with a putative role in theses processes is mammalian ATAD3, an ubiquitously expressed protein, which was initially found in a proteomic screen of rat liver mitochondria (e.g. TOB3 [6]) and later implicated as a putative marker protein in certain types of cancer [7]–[9]. Apart from its mitochondrial localization and its role in cell proliferation not much was known about the function until recent studies demonstrated the association with the mitochondrial inner membrane and an apparent role in nucleoid organization [10], [11].

Nucleoids are protein-mtDNA macrocomplexes, which are named by analogy to bacterial chromosomes. They contain about 2–8 mtDNA molecules and are organized as discrete punctae located at regular intervals throughout the mitochondrial network [12]. To date, several nucleoid-associated proteins could be identified with important functions in nucleoid organization (e.g. mitochondrial transcription factor A, twinkle helicase, mitochondrial polymerase gamma, and mitochondrial single-stranded binding protein). However, in contrast to nuclear chromatin, there is less information on the dynamics of mtDNA nucleoids. Recent studies suggest that also ATAD3 may mediate important signaling events at the outer layer of the nucleoids; however, the exact mechanism is still unresolved [11].

Here, we characterized ATAD-3, the *Caenorhabditis elegans* (*C. elegans*) homologue of vertebrate ATAD3. We demonstrate that ATAD-3 is a mitochondrial protein and RNA mediated interference (RNAi) induced depletion leads to severe phenotypes, characterized by early larval arrest, gonadal dysfunction, reduced intestinal fat storage and embryonic lethality. Investigation of mitochondrial physiology revealed a disorganized organellar morphology in young adults. Nevertheless, enzymatic measurements after *atad-3(RNAi)* demonstrated that arrested L1 larvae had similar OXPHOS activities when compared to wild type L1 larvae. However, we also observed a clear increase in OXPHOS activities at later developmental stages in wild type animals, indicating an activation of mitochondrial biogenesis, which is apparently disturbed in *atad-3(RNAi)* animals.

## Results

### F54B3.3 encodes the *C. elegans* homolog of human ATAD3

**Figure 1:**
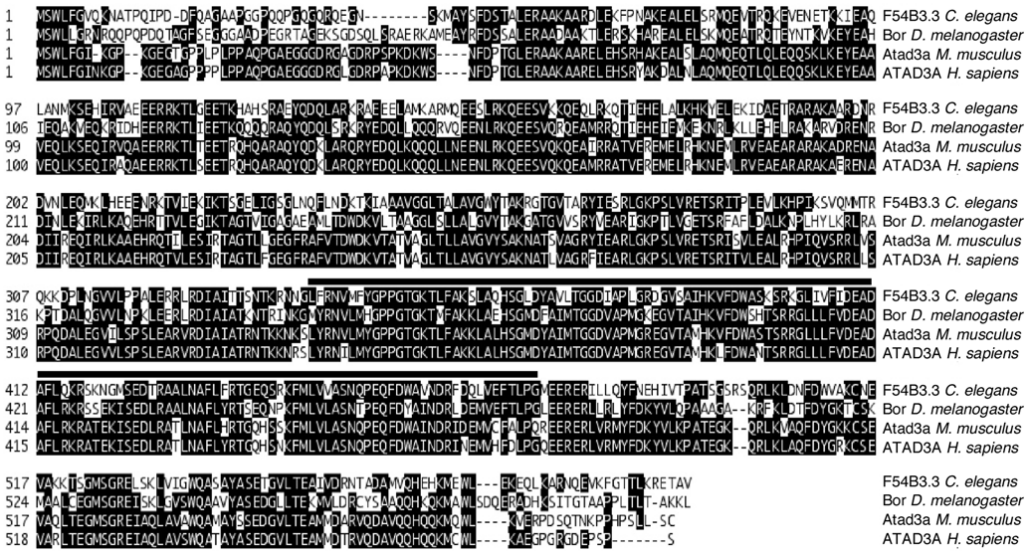
Sequence alignment of *C. elegans* F54B3.3 (ATAD-3) and predicted homologues in Drosophila (Bor), mouse (Atad3) and human (ATAD3A). Putative homologues of *C. elegans* ATAD-3 (F54B3.3) were identified by BLAST analysis ([14]). F54B3.3 encodes a protein that only contains an AAA-domain (pos. 341–474, black bar; http://smart.embl-heidelberg.de/). The sequence identity of the *C. elegans* ATAD-3 (NP_496210) AAA-domain (**A**TPases **a**ssociated with a variety of cellular **a**ctivities) compared to *Drosophila* Bor (acc. no. NP_524996), mouse Atad3a (acc. no. NP_849534) and human ATAD3A (acc. no. AAH07803) is 53%, 55% and 58%, respectively. Identical amino acid residues are highlighted in black.

Human ATAD3 is a mitochondrial protein, which is thought to be involved in mitochondrial nucleoid organization [10], [13]. Recent studies revealed that its expression is upregulated in certain types of cancer [7]–[9]. Apart from these observations, ATAD3 physiological function remains elusive. We identified a *C. elegans* protein, encoded by the predicted open reading frame (pORF) F54B3.3, as a highly conserved homologue of human ATAD3. BLAST analysis [14] revealed well conserved homologues of F54B3.3 in human (AAH07803), mouse (NP_849534) and *Drosophila* (CG6815-PA) with overall sequence identity of 58%, 55% and 53%, respectively (Fig. 1, black boxes). The sequence similarity was even higher with 77%, 73% and 73%, respectively. To ensure that F54B3.3 encodes the only *C. elegans* protein with high homology to ATAD3, we performed a BLAST search against the *C. elegans* proteome by using ATAD3 sequence as a query (http://www.wormbase.org, WS190; [15]). No other protein displayed high sequence similarity and domain composition to ATAD3. We therefore conclude that the pORF F54B3.3 encodes the *C. elegans* ATAD3 homologue and we will further refer to this gene as *atad-3*. Regarding functional domains, the AAA (ATPases associated with various cellular activities) motif [16] is highly conserved in *C. elegans* ATAD-3 (Fig. 1, black bar). Within this domain all homologues (Fig. 1) show the amino acid sequence –Asp–Glu–Ala–Asp–, which might constitute a DEAD-box motif [17]. DEAD-box proteins are involved in RNA processing, but the function of this domain has not yet been analyzed in ATAD3 homologues.

### *C. elegans* ATAD-3 is a mitochondrial protein and its depletion leads to larval arrest with low mitochondrial activity

**Figure 2:**
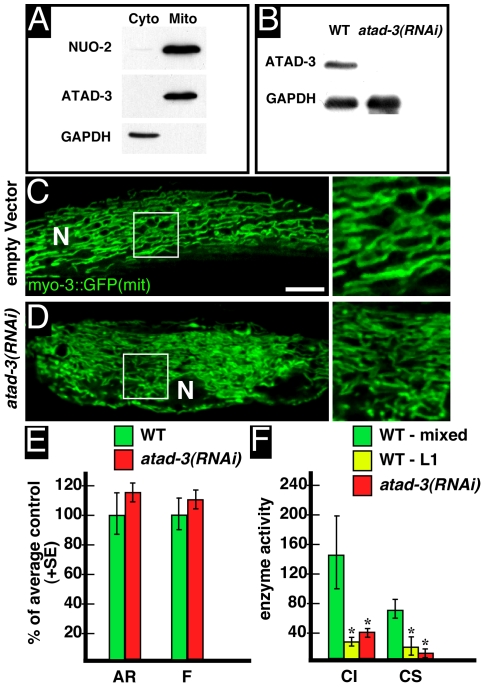
ATAD-3 is a mitochondrial protein and its depletion leads to arrest at developmental stages with low mitochondrial activity. (**A**) Western Blot analysis of *C. elegans* wild type cytoplasmic and mitochondrial protein fraction using anti-NUO-2, anti-ATAD-3 and anti-GAPDH antibodies to identify mitochondrial NUO-2/ATAD-3 (NUO-2 = homologue of the human NADH ubiquinone oxidoreductase subunit NDUFS3) and cytoplasmic GAPDH (glyceraldehyde-3-phosphate dehydrogenase), respectively. (**B**) Western blot analysis of protein extracts from wild type (WT) and *atad-3(RNAi)* animals reveals a strongly reduced ATAD-3 signal. For comparison of relative protein levels, GAPDH served as a loading control. (**C,D**) Mitochondrial morphology was analyzed in transgenic strain SJ4103 expressing a mitochondrial targeted GFP under control of the muscle-specific *myo-3* promoter ([25]). Bar: 10 µm; N = nucleus. (**C**) In SJ4103 control animals (fed with HT115 bacteria carrying the “empty” L4440 “feeding”-vector), mitochondria in body wall muscle form a well organized interconnected reticulum. (**D**) After *atad-3(RNAi)*, mitochondria appear disorganized and thinner. (**E**) Quantification of mitochondrial filamentation (aspect ratio = *AR*) and mitochondrial branching (formfactor = *F*) revealed no significant changes. Number of individual animals analyzed: WT = 11, *atad-3(RNAi)* = 11; number of objects analyzed: WT = 780, *atad-3(RNAi)* = 797. (**F**) Enzymatic activities (nmol/min/mg) of complex I (CI) and citrate synthase (CS) of a mixed stage WT population (green), WT L1 larvae (yellow) and *atad-3(RNAi*) animals (red). Asterisks indicate significant differences (p<0.05) in comparison to the control.

To confirm that ATAD-3 is indeed a mitochondrial protein, we generated anti-ATAD-3 antibodies (see Mat. & Meth.) and performed western blot analysis of *C. elegans* homogenates after separation of mitochondrial and cytoplasmic fractions (see Mat. & Meth.). As illustrated by Fig. 2A, ATAD-3 antibodies recognized a single band at approximately 70 kD in the mitochondrial fraction, which is close to the predicted size of ATAD-3 protein (67.1 kD; http://www.wormbase.org). However, there was no detectable signal in the cytoplasmic fraction. As further depicted in Fig. 2A, the anti-NUO-2 (homologue of the human NADH ubiquinone oxidoreductase subunit NDUFS3) and anti-GAPDH (glyceraldehyde-3-phosphate dehydrogenase) antibodies, serving as internal mitochondrial and cytoplasmic controls, respectively, clearly detected one band, each in the relevant fractions. Taken together, these findings demonstrate that, beside high sequence similarity between *C. elegans* ATAD-3 and human ATAD3, both protein localize to mitochondria.

To further investigate the function of ATAD-3 in *C. elegans*, we performed RNA mediated interference (RNAi) by “feeding” [18]–[20]. Western blot analysis of wild type and *atad-3(RNAi)* animals revealed a clear decrease in the level of protein expression (Fig. 2B).

Because of the mitochondrial localization of ATAD-3 and a putative role in nucleoid maintenance (see introduction), we next investigated its role for mitochondrial function. Altered mitochondrial function may be associated with changes in mitochondrial morphology in *C. elegans* [21]–[24]. Therefore, we investigated the shape of the mitochonrial network by using confocal microscopy of SJ4104 worms, carrying a mitochondrial targeted GFP [25]. As depicted in Fig. 2 *atad-3(RNAi)* animals showed a slightly disorganized mitochondrial network in comparison to control animals fed with HT115 bacteria carrying the “empty” L4440 “feeding”-vector (Fig. 2C, D; judged by three independent investigators MH, WK and FD). Moreover, mitochondria appeared to be thinner than in control animals. Computer-assisted quantification of mitochondrial branching (formfactor = *F*) and mitochondrial filamentation (aspect ratio = *AR*) of mitochondrial regions revealed a slight trend towards a more filamentous network in *atad-3(RNAi)* animals (for technical details see [26]). However, using this approach, changes were not statistically significant (Fig. 2E).

To gain information on potential changes in mitochondrial mass in young adult worms, we performed Western blot analysis of ATAD-3, NUO-2 and GAPDH protein expression levels. Experiments revealed no drastic changes in NUO-2 protein levels (as a marker of mitochondrial mass) in *atad-3(RNAi)* animals (see Fig. S1). Moreover, data obtained on the number of mitochondrial objects from the morphology analysis also indicated no clear changes (data not shown). Taken together, these results suggest no clear effect of *atad-3(RNAi)* on mitochondrial mass in adult worms/at this developmental stage.

To further investigate whether changes in mitochondrial architecture were subject of disturbed mitochondrial function or respiratory chain content, we performed enzymatic measurements of mitochondrial NADH-ubiquinone oxidoreductase (complex I) and citrate synthase (CS) in *C. elegans* tissue homogenates. Mitochondrial complex I is the largest enzyme of the respiratory chain and constitutes the entry point of electrons into the system. Cirate synthase is a key enzyme of the krebs cycle and previous studies showed that it can be used as an accurate marker of mitochondrial mass in tissue [27]. Experiments revealed a drastic reduction in complex I activity that was explained by a similar decrease in CS activity after *atad-3(RNAi)* treatment (Fig. 2F). However, we also noted that *atad-3(RNAi)* animals did not develop normally but arrested at early larval stages, primarily at the L1 stage. To study the link between CI and CS activities with the stage of development we performed enzymatic measurements on synchronized wild type L1 larvae compared to a mixed stage wild type population. It revealed that wild type L1 worms typically presented low activities of complex I and citrate synthase per milligram of tissue protein, as compared to a mixed stage wild type population. This demonstrates that ATAD-3 deficiency does not alter mitochondrial function in L1 larvae, but suggests that it hinders the transition from L1 stage to later stages where mitochondrial activity is normally enhanced. Accordingly, the drastic increase observed in mitochondrial CI and CS activities at higher larval stages (Fig. 2F) indicates that OXPHOS activity is tightly controlled and stimulated during further *C. elegans* development. These data suggest that *atad-3(RNAi)* worms cannot perform this transition.

### RNA mediated interference against *atad-3* leads to larval arrest, sterility and embryonic lethality

**Figure 3 pone-0007644-g003:**
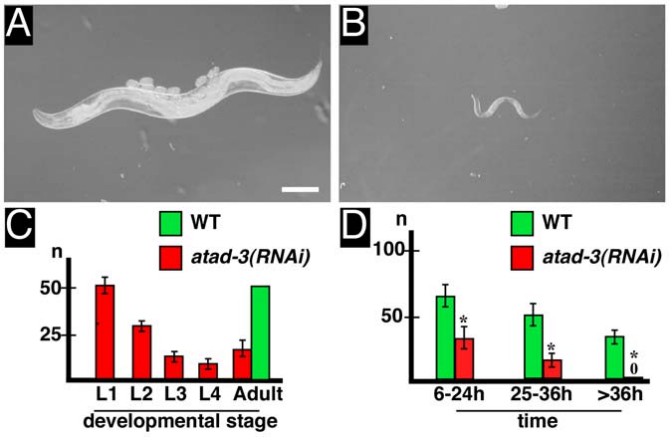
Depletion of ATAD-3 affects larval growth and fertility. (**A,B**) Nomarski optics showing adult *C. elegans* wild type (WT) animal (A) and arrested *atad-3(RNAi)* L1 larvae (B, after 72 h on RNAi “feeding”-plates). Bar (A): 100 µm. (**C**) Arrest of *atad-3(RNAi)* animals (red) in different developmental stages (L1–L4 larvae or adult) in comparison to WT (green). (**D**) Number of progeny produced by young adult hermaphrodites incubated for different times at room temperature either on *atad-3(RNAi)* “feeding”-plates (red) or control plates with HT115 bacteria carrying the “empty” L4440 “feeding”-vector (green). Asterisks indicate significant differences (p<0.05) in comparison to the control.

Based on the above mentioned observations, we investigated the phenotype caused by *atad-3 (RNAi)* in more detail. Our first observation was that most of *atad-3(RNAi)* L1 larvae remained small and showed developmental arrest at this stage compared to wild type animals (Fig. 3A–C). After incubation of 129 L1 larvae on *atad-3(RNAi)* plates for 3 days, only 20 became adult, while 11, 16, 31 and 51 arrested in L4, L3, L2 and L1 larval stages, respectively (Fig. 3C). Interestingly, the overall lifespan of *atad-3(RNAi)* animals was comparable to the control (data not shown).

When we placed young adults on *atad-3(RNAi)* plates, we first noticed a decrease in the reproduction rate. Whereas the wild type control produced about 62 embryos within in the first 12 h, *atad-3(RNAi)* animals only produced 29 embryos (Fig. 3D). During further development, the wild type reproduction rate slightly decreased over time. However, in comparison, *atad-3(RNAi)* induced a very drastic reduction, finally leading to a complete failure of reproduction after 36 h (Fig. 3D). Embryos that were produced during the first 24 h after *atad-3(RNAi)* treatment of young adults developed apparently normal but showed arrest as L1 larvae. In contrast, *atad-3(RNAi)* embryos produced by young adults between 24 h–48 h showed an embryonic lethal phenotype with failure of pronuclear migration and abnormal spindle orientation (data not shown; see also [28]).

**Figure 4 pone-0007644-g004:**
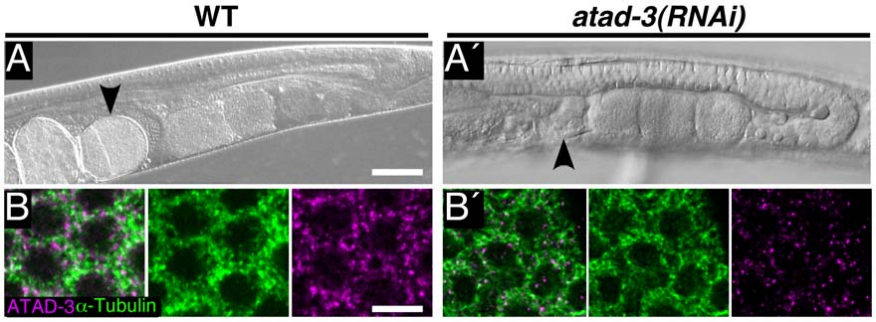
Depletion of ATAD-3 causes sterility. (**A,A′**) Nomarski optics of (A) wild type (WT) and (A′) *atad-3(RNAi)* animals showing U-shaped gonad and part of the uterus. In WT the uterus is filled with embryos (arrowhead), which are missing after depletion of ATAD-3 (arrowhead). Bar: (A) 50 µm. (**B,B′**) Confocal microscopy of oogonia in the distal part of the gonad showing immunofluorescence of ATAD-3 (red) and α-Tubulin (green) and in WT (B) and after *atad-3(RNAi)* (B′). Bar: (B) 10 µm.

In *atad-3(RNAi)* adults (see above), the overall gonade morphology, as judged by light microscopy, appeared to be rather normal, containing gonadal arms with a distal region, a loop and a proximal region where oocytes are formed (Fig. 4A, A′). In *atad-3(RNAi)* gonads the anti-ATAD-3 signal was strongly reduced but oogonia retained a rather well organized honeycomb-like pattern (Fig. 4B, B′). However, after depletion of ATAD-3 oocytes became not fertilized and embryos did not show up in the uterus as in wild type (compare Fig. 4A and Fig. 4A′ [arrowheads] ), thus leading to sterility.

### RNA mediated interference against *atad-3* reduces intestinal fat content

**Figure 5:**
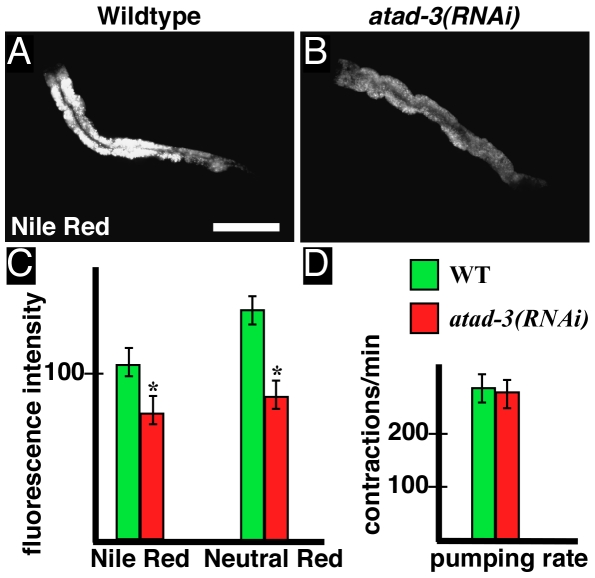
Comparison of intestinal fat storage and lysosomal content in wild type (WT) and *atad-3(RNAi*) hermaphrodites. (**A,B**) Visualization of intestinal lipid droplets by Nile Red staining in (A) living WT and (B) *atad-3(RNAi)* animals. Bar (A): 100 µm. (**C**) Quantification of Nile Red and Neutral Red (lysosomal marker) fluorescence intensity (relative pixel intensity) in WT (green) and *atad-3(RNAi)* animals (red). (**D**) The pharyngeal pumping rate of adults (as indicated in contractions per minute) is not significantly altered after *atad-3(RNAi)*. Asterisks (C) indicate significant differences (p<0.05) in comparison to the control.

The intestine is a main organ of energy generation in *C. elegans* [29]. Intestinal fat storage and lysosomal content of *C. elegans* are influenced by metabolic activity, feeding behaviour and gonadal function [30]. Importantly, it has been described that mitochondrial oxidative phosphorylation is essential for intestinal lipid accumulation [31]. Therefore, these parameters appeared to be of special interest in our *atad-3(RNAi)* analysis. Both parameters can be measured by the vital dyes Nile Red (lipid droplet staining; [32]) and Neutral Red (lysosome staining), followed by video-imaging and fluorescence microscopy [33]. Experiments revealed that *atad-3(RNAi)* animals accumulated significantly less fat than their wild type counterparts (Fig. 5A–C). Moreover, neutral red staining indicated a reduced lysosomal content in *atad-3(RNAi)* animals (Fig. 5C). Of note, the observed alterations were not caused by a disturbed feeding behavior as indicated by measurements of pharyngeal pumping rate in young adults (Fig. 5D).

## Discussion

Cell proliferation is an energy consuming process that is controlled by checkpoints of the cell cycle machinery. Dividing cells must be capable of dealing with the structural and energetic demands imposed by proliferation. Changes in mitochondrial number and morphology together with the replication and redistribution of mtDNA are critically involved in these processes [34]. The proposed role of ATAD3 as a regulator of nucleoid formation and/or segregation is in accordance with the observed alterations in cell division after knock-down of ATAD3 [8], [9] and receives additional importance with regard to high protein expression levels in certain types of cancer. However, the role of ATAD3 during development of a multicellular organism has not been addressed so far.

Our data clearly indicate that in *C. elegans* ATAD-3 is essential for larval development and required for proper organ function. In view of the mitochondrial localization and a putative role in nucleoid organization (Fig. 2), defects in mitochondrial function and/or biogenesis might be suspected as the underlying cause for the observed defects in cellular proliferation and metabolic activity (Figs. 2–5) after depletion of ATAD-3. Systematic RNAi screens in *C. elegans* revealed a crucial role for mitochondria in fertility and development [21]. Several detailed investigations of mitochondrial dysfunctions have been described in *C. elegans* so far [22]–[24], [35], [36]. Key abnormalities that were observed in these different studies included reduced fertility (including gonadal dysfunction), impaired development and slow growth. In addition, alterations in mitochondrial morphology have been reported, ranging from severe fragmentation to increased filamentation [22], [24].

In a combined view of these findings, interesting similarities to our *atad-3(RNAi)* phenotype arise. Apart from the severe developmental disturbance, the observed alterations in mitochondrial organization are interesting and might indicate a functional defect. Increasing evidence has been provided that mitochondria form a functional reticulum, which on the one hand protects individual mitochondria from stochastic depletion of metabolic substrates or mtDNA and on the other hand mediates the sharing/transport of intramitochondrial constituents like antioxidants [37]–[39]. Therefore, changes in mitochondrial network organization, as observed in polymerase gamma deficient *C. elegans* [24] or after depletion of ATAD-3, might indicate a compensatory strategy to counteract problems concerning mitochondrial function or mtDNA maintenance. In addition, also cell cycle defects or cellular senescence may be associated with changes in mitochondrial architecture and increased branching, at least in human cell culture systems [40]. The observation that mitochondria appeared slightly thinner in *atad-3(RNAi)* animals may indicate additional changes in mitochondrial architecture. However, at the current stage, we cannot rule out that these changes may be due to reduced GFP transport to the mitochondrial matrix.

A remarkable finding of our study was that *C. elegans* L1 larvae displayed very low complex I and citrate synthase activities when compared to a mixed stage wild type population, indicating a low mitochondrial content at this developmental stage. Apparently, *atad-3(RNAi)* animals mainly arrest at developmental stages with low mitochondrial content. These findings indicate that OXPHOS derived energy requirements are rather low during early larval development, whereas during later development the activation of OXPHOS/biogenesis of mitochondria is initiated to fulfill metabolic demands. These observations are in keeping with other studies showing that mtDNA copy number drastically increases especially after the L3 stage [24]. Of note, also studies in humans described a marked increase of OXPHOS activities and mitochondria content in postnatal compared to prenatal tissues, which indicates a more general phenomenon behind these observations [41]. Apparently, *atad-3(RNAi)* worms mainly arrest at developmental stages with low mitochondrial tissue content and activity.

The most obvious tissue-specific dysfunction that we observed after *atad-3(RNAi)* was a failure of fertilization. The distal part of the *C. elegans* gonad is a non-cellularized syncytium where germ line nuclei divide, while in the proximal part cellularization and oocyte growth takes place [42]. Because the gonad is densely packed with mitochondria and constitutes the primary site of mtDNA replication in *C. elegans*, alterations in mitochondrial function might disrupt normal tissue function [24]. Results from several studies suggest that gonadal development strongly depends on proper mitochondrial biogenesis and function [22], [43], [44]. Nevertheless, sterility could also arise due to defects in the germ cells [45], in the somatic gonad [46] or in the spermatheca epithelium [47] . After RNAi against *atad-3* no obvious defects became apparent in the distal and proximal region of the gonad, as judged by immunofluorescence and Nomarski optics, respectively (Fig. 2). A further detailed analysis is necessary to uncover the exact nature of the sterility phenotype. In general, these observations might indicate that tissues with high mitotic and metabolic (mitochondrial) activity are especially vulnerable to ATAD-3 deficiency.

In line with this idea, we also observed a reduced intestinal fat storage and lysosomal content in young adults after *atad-3(RNAi)*. These findings might suggest a disturbed balance between fat storage and mobilization, possibly indicating a problem with energy homeostasis and metabolic activity. Importantly, McKay *et al*. (2003) described that disturbed function of the mitochondrial respiratory chain severely impairs intestinal fat storage in *C. elegans* [31]. The existence of an endocrine signaling axis might link crucial organ systems in *C. elegans* (e.g. the germ line and the intestine [30]) and hence would explain the coincidence of observed gonadal and intestinal phenotypes after *atad-3(RNAi)*.

Although our *atad-3(RNAi)* analysis reveals interesting parallels to phenotypes caused by other mitochondrial dysfunctions in *C. elegans* (see introduction), the bioenergetic problem might not to be the sole explanation concerning the very early larval and embryonic arrest. So far, the genetic control of the progression through larval stages in *C. elegans* is still not fully understood. Development and growths certainly require a large number of regulatory factors that orchestrate cell division and tissue differentiation. Apparently, ATAD-3 could be involved in these processes too. Data from human cell culture systems showed that ATAD3A mRNA is especially present in regenerating tissues [9]. ATAD3A deficiency might lead to cell cycle arrest with polynuclear cells, finally causing apoptosis [8].

In summary, our data clearly indicates that ATAD-3 is essential for *C. elegans* development *in vivo*. Moreover, our results suggest that the protein is crucial for the upregulation of mitochondrial activity during the progression through larval stages. It will be an important future task to further investigate the exact function of ATAD-3 during *C. elegans* development and to elucidate the mechanisms underlying the abnormalities that we observed in ATAD-3 deficient worms.

## Materials and Methods

### Strains

Maintenance and handling of *C. elegans* were carried out as described previously [48]. Bristol N2 was used as the wild type strain. Transgenic SJ4103 strain carries a mitochondrial targeted GFP under the control of the *myo-3* promoter [25].

### ATAD-3 antibody production

PCR was used to amplify a 900 base pair fragment, encoding the C-terminal 300 amino acids of ATAD-3 (-HPIK… ETAV): forward: 5′-GGGGATCCCACCCAATTAAAAGTGTTCAAATGATG-3′, reverse: 5′-GGGGTACCTTAAACAGCAGTTTCTCTCTTCAACGT-3′ (synthetic restriction sites for *Bam*HI and *Kpn*I are underlined). The fragment was cloned *in frame* in the 6× His-tag expression vector pQE30 (Qiagen), transformed and induced in *E. coli* strain M15 [pREP4] (Qiagen). Recombinant protein was purified on Ni**^2+^**-NTA matrix (Qiagen) and sent to Eurogentec (Seraing, Belgium) for immunization. The final bleed of one rabbit was purified using columns and the recombinant protein as a bait. For elution of anti-ATAD-3 antibodies Tris-Cl (pH 8.0) was used.

### RNA-mediated interference (RNAi)

RNAi by “feeding” was performed essentially as described by [20], (“Protocol I”). The RNAi clone for *atad-3* was obtained from the Ahringer RNAi library (Geneservice Limited). After amplification of a single colony overnight (37°C, LB amp tet medium), HT115 (DE3) bacteria (RNase III-deficient *E. coli* strain, carrying IPTG-inducible T7-polymerase) were seeded on NGM_amp_tet_ plates, containing IPTG (1 mM) and further incubated overnight at room temperature (22°C) to allow the expression of double-stranded RNA. HT115 (DE3) bacteria harboring the “empty” KS+based vector, L4440 (containing two T7 promoters flanking a polylinker), were used as a control for RNAi “feeding” experiments.

### Larval phenotype and embryonic production

To determine the larval arrest phenotype, wild type L1 stage larvae were either placed on plates seeded with HT115 bacteria containing the “empty” feeding vector L4440 (“WT control”) or on *atad-3(RNAi)* bacteria plates. The plates with worms were incubated at room temperature and evaluated after 3 days. The effects on embryonic production were determined by counting the numbers of progeny that managed to hatch on either “WT control” plates or on *atad-3(RNAi)* bacteria plates.

### Immunostaining

Gravid adults were transferred with a drawn-out pipette to a microscope slide, coated with a thin layer of polylysine in a drop of sterile M9 buffer and cut with a scalpel. Embryos were immediately permeabilized by the freeze-crack method [49] and fixed in 100% methanol (10 min), 100% acetone (20 min), 90% ethanol (10 min), 60% ethanol (10 min), and 30% ethanol (10 min). Slides were washed twice for 10 min each with TBT [Tris-buffered saline (25 mM Tris), plus 0.1% Tween 20 or Triton X-100], incubated at 4°C overnight with primary antibodies (see below) in blocking buffer (TBT plus 1% bovine serum albumin and 1% nonfat dry milk powder), washed three times for 10 min each with TBT at room temperature and incubated at room temperature for 1–3 h, with secondary antibodies (see below) in blocking buffer. Finally, slides were washed three times for 10 min each in TBT and mounted in Mowiol containing 1,4-diazabicyclo(2.2.2)octane (Sigma-Aldrich) as an antifade reagent. To stain gonads, young adults were cut in M9 buffer with a scalpel on a polylysin coated slide, fixed in paraformaldehyde for 1 h at room temperature, washed briefly with TBT, incubated for 1.5 h at room temperature with primary antibodies, washed twice for 10 min each with TBT, and incubated with secondary antibodies for 1 h. The following primary and secondary antibodies were used at the dilutions (in blocking buffer) indicated: anti-ATAD-3 (rabbit, 1∶200, see below), anti-a-Tubulin (mab4A1, mouse, 1∶40, [50]). Secondary antibodies were Cy2 and Cy3 (1∶200; Jackson Immunoresearch Laboratories, West Grove, PA). Analysis of immunofluorescence was performed on a confocal laser-scanning microscope (LSM 510 Meta, Zeiss).

### Video imaging and confocal laser-scanning microscopy in living worms

For microscopy analysis, animals were mounted on 2% agarose pads and immobilized with levamisole (200 µM). For phenotypic characterization, worms were investigated under a Zeiss Axioplan 2 research microscope fitted with a Hamamatsu Orca-ER camera. To analyze mitochondrial structure, muscle cells of SJ4103 young adults [25] were investigated by confocal laser-scanning microscopy (LSM 510 Meta, Zeiss; Plan-Apochromat 63x/1.4 oil DIC; 1468×1468 pixel). Mitochondrial filamentation (aspect ratio = *AR*) and mitochondrial branching (formfactor = *F*) were quantified by using a modified computer-assisted approach published by Koopman *et al*., 2008 (for technical details see [26]). In each experiment, the average value obtained with control cells was set at 100%, to which all other values were related. Values from multiple experiments were expressed as means±SEM. Statistical significance (Bonferroni corrected) was assessed using Student's t-test.

### Immunoblotting

Western blot analysis was performed essentially as described previously [51]. In brief, animals were collected in M9 buffer, frozen in liquid nitrogen and boiled in SDS sample buffer für 10 minutes. Samples were loaded on a 10% SDS polyacrylamide gel and blotted on a nitrocellulose membrane. Detection was performed using BM chemiluminescence substrate (Boehringer Roche Diagnostics) and secondary antibodies (HRP-conjugated, 1∶10000). The following primary antibodies were used: anti-ATAD-3 (rabbit, 1∶1000), anti-NUO-2 (MS112, mouse, 1∶2000, MitoSciences), anti-GAPDH (AM4300, mouse, 1∶2000, Ambion).

For separation of mitochondrial and cytoplasmic fractions, worms were harvested from well grown NGM plates and washed twice with M9 buffer. Washed worms were suspended in MSE buffer (25 mM Mannitol, 75 mM Sucrose, 0.1 mM EDTA, pH 7.4). After freeze/thaw three times using liquid nitrogen, worms were homogenized at 4°C, using a glass homogenizer and centrifuged at 4°C, 2000 rpm for 15 minutes. To separate mitochondria from cytoplasmic fraction the supernatant was centrifuged at 4°C, 13000 rpm for 15 minutes (pellet = mitochondria, supernatant = cytoplasmic fraction).

### Enzymatic NADH ubiquinone oxidoreductase and citrate synthase activity measurements

Animals were collected in M9 buffer and frozen in liquid nitrogen. Semi-synchronized L1s were obtained by treatment with alkaline hypochloride (2 vol 4 M NaOH : 3 vol 13% NaOCl) ([52]). The pellet was suspended in homogenizing buffer (20 mM TrisCl pH 7.2, 250 mM sucrose, 40 mM KCl, 2 mM EGTA, 1 mg/ml BSA) using a glass homogenizer. The resulting homogenate was centrifuged at 500×g for 20 min at 4°C. The pellet was resuspended in homogenizing buffer and centrifuged at 650×g for 20 min at 4°C. The supernatants of both centrifugation steps were combined and frozen in liquid nitrogen until use. Assays of respiratory chain enzyme activities were carried out spectrophotometrically on a double wavelength UVMC^2^ spectrophotometer (SAFAS), using standardized and reproducible methods as previously described [27]. All activities are expressed in nmol/min/mg. The oxidation of NADH by complex I (NADH ubiquinone oxidoreductase) was recorded using ubiquinone as electron acceptor. The basic assay medium (35 mM KH_2_PO_4_, 5 mM MgCl_2_, 2 mM KCN, pH 7.2) was supplemented with 2.5 mg/ml BSA, 2 µg/ml antimycin A, 0.1 mM UQ1decylubiquinone and 0.1 mM NADH in a final volume of 1 ml. Enzyme activity was measured by starting the reaction with 20 µg of mitochondrial protein. The decrease in absorption due to NADH oxidation was measured at 340 nm in both the absence and presence of rotenone 10 µg/ml. The difference in both activities indicates the rotenone-sensitive activity of complex I. The extinction coefficient used for NADH was 6.22 mM^−1^ cm^−1^. The reduction of 5′,5′-dithiobis(2-nitrobenzoic acid) (DNTB) by citrate synthase (CS) at 412 nm (extinction coefficient 13.6 mM^−1^ cm^−1^) was followed in a coupled reaction with coenzyme A and oxaloacetate. A reaction mixture of 20 mM Tris-HCl, pH 8.0, 0.42 mM acetyl-coenzyme A, 0.1 mM DTNB and 20 µg of mitochondrial proteins was incubated at 37°C for 5 min. The reaction was initiated by the addition of 0.5 mM oxaloacetate and the absorbance change monitored for 5 min.

### Nile Red and Neutral Red experiments

Assays were performed essentially as described by others [32], [33]. In brief, young adults were placed on OP50 bacteria containing Neutral Red or Nile Red, respectively. Intensity was monitored by fluorescence microscopy and quantified by using ImageJ software (developed by Wayne Rasband at the National Institutes of Health, available at http://rsbweb.nih.gov/ij/).

## Supporting Information

Figure S1 Western blot analysis of ATAD-3, NUO-2 and GAPDH protein expression levels. Western blot analysis of ATAD-3, NUO-2 and GAPDH protein expression levels in young adult WT and atad-3(RNAi) animals. Experiments revealed no drastic changes in NUO-2 protein levels in atad-3(RNAi) animals, suggesting no major influence of atad-3(RNAi) on mitochondrial mass in these worms/at this developmental stage.
(0.85 MB TIF)
