# Supplementary figures and images for: C. elegans ATAD-3 Is Essential for Mitochondrial Activity and Development
Source: PLoS One. 2009 Oct 30;4(10):e7644. doi: 10.1371/journal.pone.0007644 (PMC2765634; doi:10.1371/journal.pone.0007644)

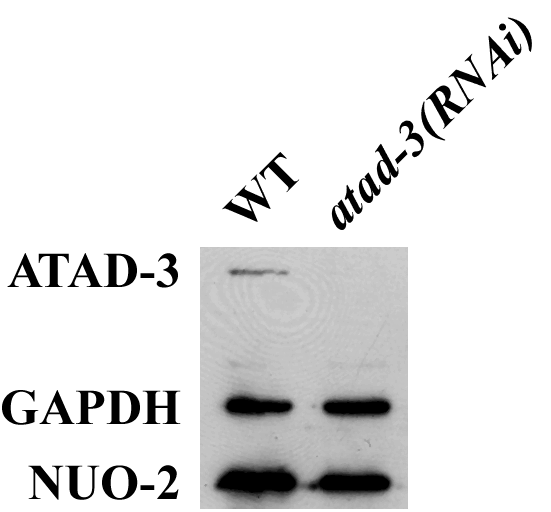

Supplement: Figure S1 — Western blot analysis of ATAD-3, NUO-2 and GAPDH protein expression levels. Western blot analysis of ATAD-3, NUO-2 and GAPDH protein expression levels in young adult WT and atad-3(RNAi) animals. Experiments revealed no drastic changes in NUO-2 protein levels in atad-3(RNAi) animals, suggesting no major influence of atad-3(RNAi) on mitochondrial mass in these worms/at this developmental stage. (0.85 MB TIF) [file pone.0007644.s001.tif]
